# Ethnic differences in association of high body mass index with early onset of Type 1 diabetes – Arab ethnicity as case study

**DOI:** 10.1371/journal.pone.0175728

**Published:** 2017-04-13

**Authors:** Arshad M. Channanath, Naser Elkum, Dalia Al-Abdulrazzaq, Jaakko Tuomilehto, Azza Shaltout, Thangavel Alphonse Thanaraj

**Affiliations:** 1 Dasman Diabetes Institute, Dasman, Kuwait; 2 Department of Pediatrics, Faculty of Medicine, Kuwait University, Safat, Kuwait; Baylor College of Medicine, UNITED STATES

## Abstract

**Objective:**

The “accelerator hypothesis” predicts early onset of Type 1 diabetes (T1D) in heavier children. Studies testing direction of correlation between body mass index (BMI) and age at onset of T1D in different continental populations have reported differing results–inverse, direct, and neutral. Evaluating the correlation in diverse ethnic populations is required to generalize the accelerator hypothesis.

**Methods:**

The study cohort comprised 474 Kuwaiti children of Arab ethnicity diagnosed with T1D at age 6 to 18 years during 2011–2013. Age- and sex-adjusted BMI z-scores were calculated by comparing the BMI measured at diagnosis with Kuwaiti pediatric population reference data recorded during comparable time-period. Multiple linear regression and Pearson correlation analyses were performed.

**Results:**

BMI z-score was seen inversely associated with onset age (r,-0.28; p-value<0.001). Children with BMI z-score>0 (*i*.*e*. BMI >national average) showed a stronger correlation (r,-0.38; p-value<0.001) than those with BMI z-score<0 (r,-0.19; p-value<0.001); the former group showed significantly lower mean onset age than the latter group (9.6±2.4 *versus* 10.5±2.7; p-value<0.001). Observed inverse correlation was consistent with that seen in Anglo-saxon, central european, caucasian, and white children while inconsistent with that seen in Indian, New Zealander, and Australian children.

**Conclusions:**

The accelerator hypothesis generalizes in Arab pediatric population from Kuwait.

## Introduction

The “Accelerator Hypothesis” [1, 2] proposes three processes that accelerate the rate of beta cell loss. The three accelerators include (i) constitutionally high rate of beta-cell apoptosis, (ii) insulin resistance, and (iii) beta cell autoimmunity. Gain of excess weight deems to be central to the rising incidence of diabetes and onset of diabetes in early age. Gain in weight increases insulin resistance and leads to poor glucose control [3]. None of the proposed three accelerators is known to be effective in the absence of weight gain.

Several studies have examined relationships between body mass index (BMI) and age at onset of Type 2 diabetes (T2D) [4–8], and these studies led to the conclusion that higher obesity levels are associated with early onset of T2D. However similar studies on Type 1 diabetes (T1D) in several populations (including United States of America, United Kingdom, Central Europe, Spain, India, New Zealand, and Australia [9–21]), revealed, unlike in the case of T2D, varied results on the direction (i.e. direct, inverse or neutral) and degree of correlations between BMI and age at onset. Populations from the Arabian Peninsula, such as that of Kuwait which has fourth highest incidence of childhood T1D in the world [22], are underrepresented in such studies.

Prevalence of childhood obesity is alarmingly high in Kuwait and exceeds the prevalence rates reported from North America [23]. High prevalence and rising incidence of childhood T1D have been reported in Kuwait [24–26] and other countries from the Arabian Peninsula [27]. The incidence of childhood-onset T1D has doubled in Kuwait over the last two decades with the recent incidence rate of 40.9 per 100000 per year in children aged 14 years or under [28,29]. Adulthood T2D is also prevalent in Kuwait [30] and the region [31,32]. The genetic makeup of this population is unique [33] and the genetic profile of diabetes in this population is probably different from that seen in western population [33–35]. Thus, testing the correlation between body mass and age at onset of T1D in Arab populations may add insights to what has been learnt from other populations on generalizing the “Accelerator Hypothesis”.

In this study, we delineate the direction (direct or inverse or neutral) and degree of relationship between BMI z-scores and age at onset of T1D in Kuwaiti pediatric population and compare with results reported from studies on global populations.

## Materials and methods

### Data repository

The Kuwait’s national Childhood-Onset Diabetes electronic Registry (CODeR) (https://coder.health.org.kw:444/index.php), maintained at Dasman Diabetes Institute, captures clinical details on Kuwaiti children diagnosed with T1D during the age of 1 to 18 years. Informed consent from parents of the children and informed assent from the children were obtained for participation in research projects. Diagnosis was established according to the guidelines issued by World Health Organization [36,37]. Date of administering the first insulin injection was considered as the date of onset. Clinicians in government hospitals throughout Kuwait manually input patient details at the time of diagnosis into the repository via a software application (using personal computer or iPad connected through a secure virtual private network). Capture of linked laboratory data is partially automated and is drawn from multiple sources and includes data validation. Data linkage is facilitated by a unique civil identifier assigned by Public Authority for Civil Information (PACI) to each of the residents in Kuwait. Notifications on children newly diagnosed with T1D from Primary Healthcare Centers and the Kuwait Diabetes Society were done through a surveillance system.

### Data extracted from repository for our study

The cohort used for the presented study comprised children from the registry diagnosed with T1D during 2011 to 2013. The extracted data included age, sex, nationality, date of birth, date of diagnosis, type of diabetes, height/length and weight measurements, family history of diabetes, levels of plasma glucose & glycated haemoglobin (HbA1c), and blood pressure readings.

### Considered subjects

**Fig 1:**
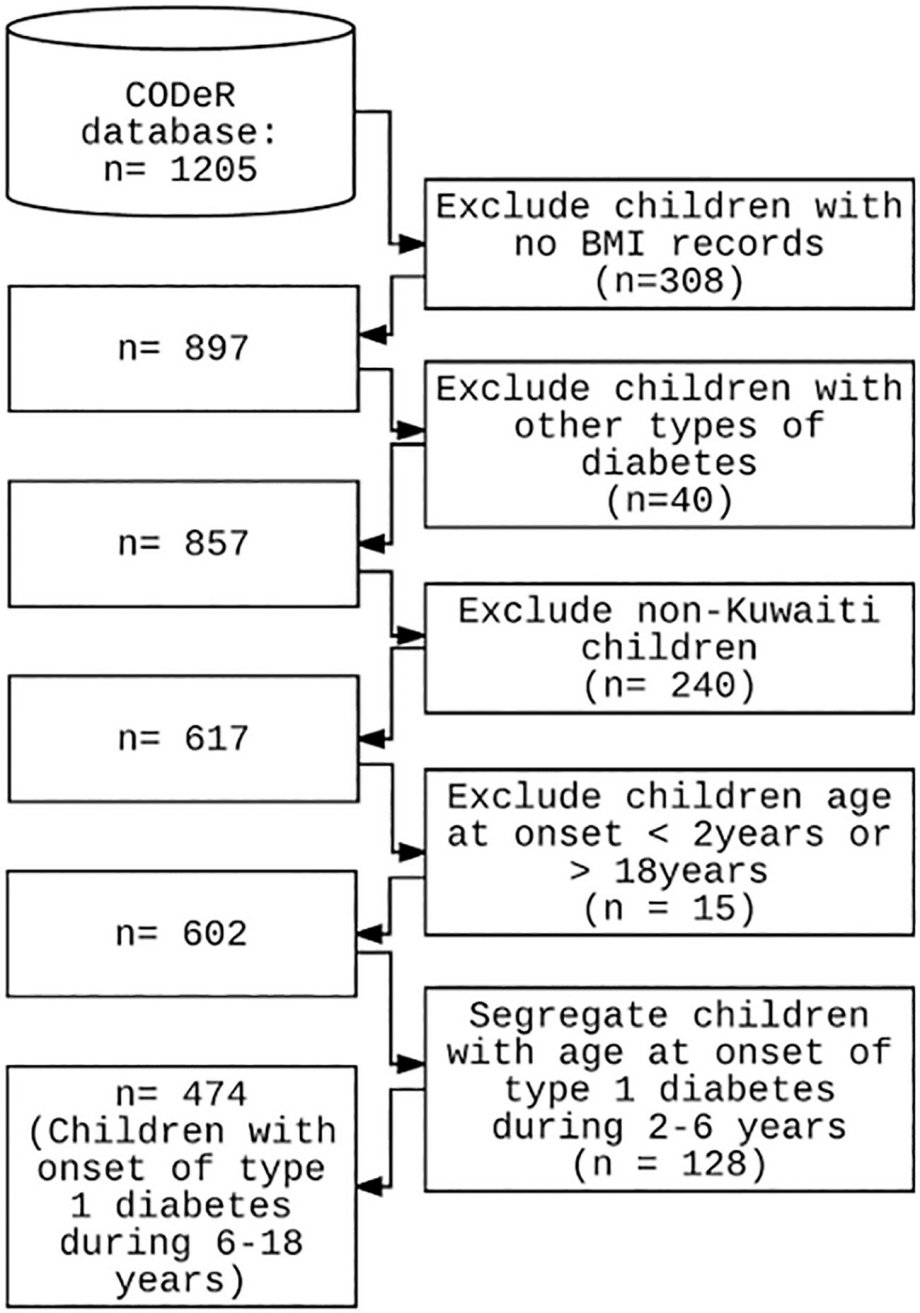
Flowchart depicting the filtering steps used to derive the data sets on children and adolescents with age at onset of T1D during 2 to <6 years, and during 6 to18 years.

The repository contained data on 1205 children and adolescents diagnosed with diabetes during 2011 to 2013. The filtering steps used to scrutinize these data for deriving the final data sets used in the study are depicted in Fig 1. The resultant participants diagnosed with T1D during the period of 2011 to 2013 were 602 native Kuwaiti children; of these 128 had onset within 2 to <6 years of age, and 474 within 6 to 18 years of age.

### National reference data on BMI for the childhood population

Reference data on BMI for children aged 6 to18 years were obtained from a cross-sectional epidemiological study on childhood obesity in the state of Kuwait [23]; the study recruited schoolchildren, of native Kuwaiti ethnicity, from all the six governorates. At the time of participant recruitment, the nationality was confirmed and data on age, sex, and illnesses (e.g. diabetes and other chronic diseases) were recorded. Anthropometric measures (height, weight, and waist circumference) and blood pressure readings were recorded. Informed consent from parents of the children and informed assent from the children were obtained for participation in research projects. From this epidemiological study, we considered 5164 healthy individuals (boys: 1985; girls: 3179) of 6 to 18 years of age recruited over a comparable time-span of 2011 to 2013 (same time-span during which the participants of the study cohort were diagnosed with T1D). For children aged 2 to <6 years, we do not have any national reference data and hence an internal reference data from CODeR was used; such a reference data comprised 71 boys and 86 girls.

### Derivation of BMI z-score

BMI measured at the diagnosis of T1D was compared with national reference data to derive age- and sex-adjusted BMI z-score. The BMI z-scores were derived using the LMS method of Box–Cox power transformation [38], which adjusts the distribution of the parameter for skewness. Relationship between BMI and age at diagnosis was examined using such BMI z-scores.

### Blood pressure and parental history of diabetes

High blood pressure was defined as systolic and/or diastolic blood pressure ≥ 95th percentile for sex and age [39]. Status of the parents who had either T1D or T2D was considered as positive parental history of diabetes.

### Statistical analysis for associations

Associations between age at onset of T1D and age- and sex-adjusted BMI z-scores at onset were evaluated using multiple linear regression model. Pearson correlation test was also used as the metric of association. The study had sufficient power to show a correlation of r = -0.28 with 80% power with 95% certainty. Multiple linear regression analysis was performed to explore the moderator effect on the association between BMI z-score and onset age (i) of being heavier than the national average; and (ii) of possessing parental history of diabetes or of being hypertensive. Results were considered statistically significant at p-values <0.05. In order to examine differences in correlations in patients with BMI above the national average *versus* those with BMI below the national average, we defined a dichotomous variable ‘FAT’, which was set = 1 for those patients with BMI z-score >0; and set = 0 for those with BMI z-score <0. Data analysis was performed using the R computing environment (R: A language and environment for statistical computing, version 3.2.0. R Foundation for Statistical Computing, Vienna, Austria. URL http://www.R-project.org/).

### Data availability

A minimal data file, listing for every considered participant of the cohort sex, BMI z-score and age at onset of T1D, is provided as S1 Dataset.

## Results

### Observations with the cohort of children diagnosed for T1D during the age of 6 to 18 years

#### Descriptive statistics on the cohort

A total of 474 Kuwaiti children (228 boys and 246 girls) with new onset of T1D during the age of 6 to 18 years were included in the study. Parental history of diabetes was found in 106 (22.4%) children and high blood pressure at the onset of diabetes was found in 26 (5.5%) children. The mean values for age at onset of T1D and BMI z-score in the cohort with onset age of 6–18 years were 10.22±2.63 years and -0.417±1.06, respectively. In our cohort, the mean age at onset in girls was seen slightly but significantly younger than that in boys (9.9 and 10.6 years, respectively; p-value = 0.006) though the difference in BMI z-scores was not statistically significant (p-value = 0.16).

#### Correlation between BMI z-score and age at onset of T1D

The results of Pearson correlation tests, that assessed the relationship between age at onset of T1D and BMI z-score, indicated that a significant linear inverse correlation (r = -0.28, p-value = 10e-07, n = 474) was found between BMI z-scores and age at onset. Sex-specific data also showed inverse relationship (r_boys_ = -0.24, p-value = 0.003, n = 228; r_girls_ = -0.30, p-value = 10e-05, n = 246).

#### Correlations in children with BMI higher versus lower than the national average

**Fig 2:**
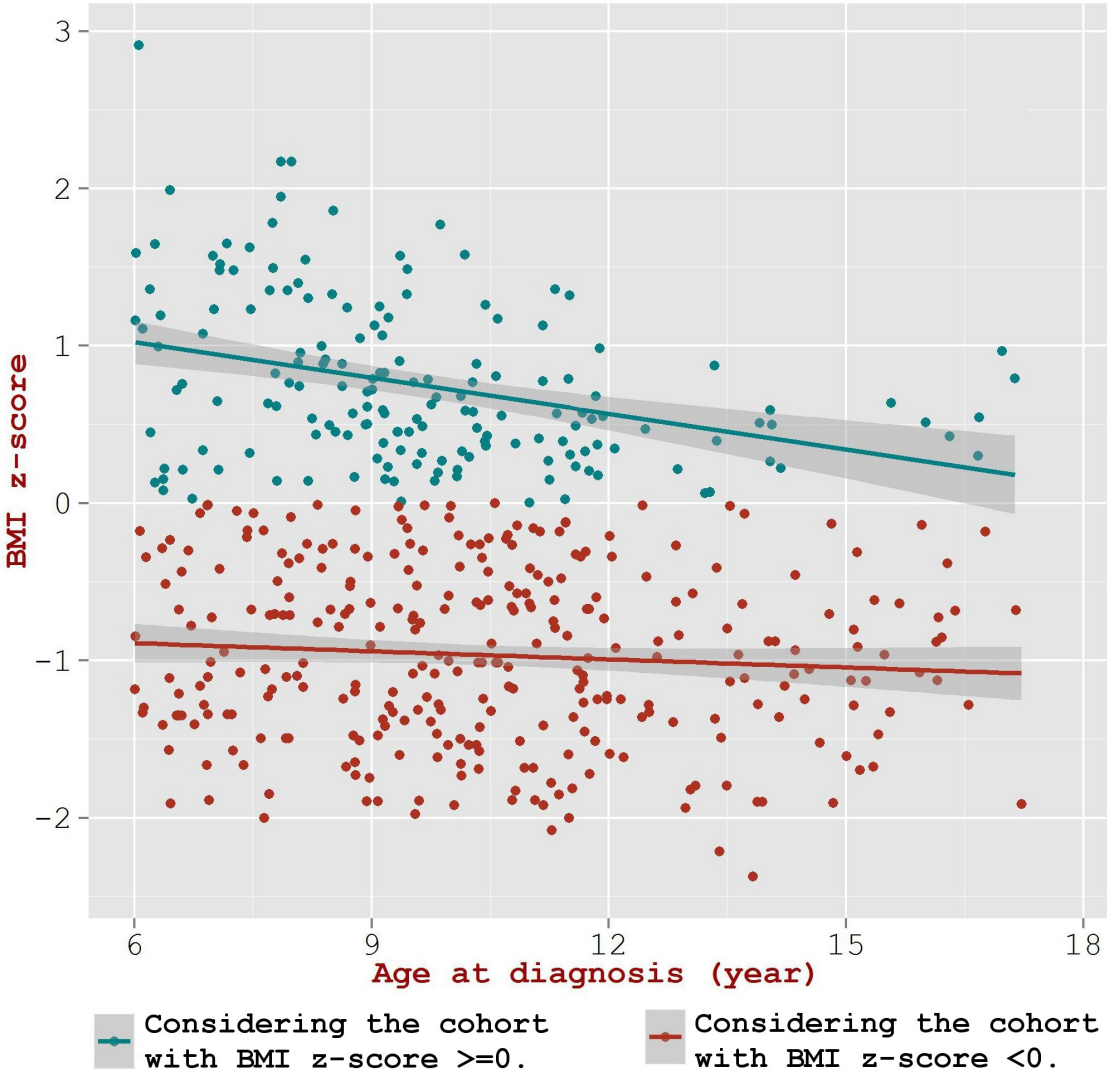
Multiple linear regression models for association between BMI z-score and age at onset of T1D in the cohort of 6 to 18 years stratified by BMI z-score >0 and <0.

Of the 474 children diagnosed for T1D during 6–18 years, 168 showed a positive BMI z-score (*i*.*e*. z-score >0) and the remaining 306 children showed a negative BMI z-score (*i*.*e*. z-score <0). The correlation coefficient in the children with positive BMI z-scores was significantly higher than that in the children with negative BMI z-scores (r_positive BMI z-score_ = -0.38; r_negative BMI z-score_ = -0.19, p-value = 0.047) (Fig 2). Upon examining the effect of higher BMI levels as a moderator of the relation between onset age and BMI z-score, we observed a statistically significant effect by body composition—as evidenced by the values (Beta = -0.85, p-value = 0.009) for the interaction term between BMI z-score and ‘FAT’ (see methods) in the regression model. Fig 2 illustrates that the points were clustered to lower onset age along the X-axis (age at onset) for the positive BMI z-score cohort, while they were dispersed along the X-axis for the cohort with negative BMI z-scores. Further, we find that the mean age at onset in children with positive BMI z-score was significantly lower than the children with negative BMI z-score (9.6±2.4 *versus* 10.5±2.7; p-value = 0.0001).

#### High blood pressure and parental history of diabetes

Further calibration of the BMI z-scores for blood pressure and parental history of diabetes still retained the significant correlation between BMI z-scores and age at onset of T1D. However, the interaction terms involving the parental history of diabetes or high blood pressure in the multivariate linear regression model did not show any statistical significance (p-value = 0.90 and 0.81, respectively).

### Cohort of children diagnosed during the age of 2 to 6 years

In children diagnosed with T1D during 2 to 6 years of age, we did not observe any significant correlation between BMI z-scores and age at onset (r = -0.029, p-value = 0.74, n = 128; r_boys_ = 0.02, p-value = 0.874, n = 60; r_girls_ = -0.07, p-value = 0.556, n = 68).

## Discussion

The ‘accelerator hypothesis’ postulates that weight-related insulin resistance accelerates the onset of T1D [1,2]. It predicts that a heavier child has the potential to develop T1D at a younger age. This prediction has been tested in various populations (Table 1) with varying results ranging from direct correlation between BMI and age at onset of T1D to no correlation and inverse correlation. While studies dealing with children from Spain [14] showed direct correlation, studies from India [10,21], New Zealand [19], and Australia [15] showed no correlation, and studies dealing with white children from USA [13,17], Central Europe [12], and United Kingdom [9,11] showed the expected inverse correlation. A need for further studies to test whether the proposed accelerator hypothesis is relevant in every ethnic population has been raised [21].

**Table 1:** Summary of findings from previous studies on association between age- and sex-adjusted BMI z-scores and age at onset of T1D in various populations.

Study	Study population	Cohort size, age at diagnosis, study period, and source of reference growth charts	Nature of correlation between BMI z-score and age at onset of T1D.
[Gimenez et al., 2007] [14]	Mediterranean–Catalan region (northeast Spain)	3203, 2 to 25 years, 1990 to 2004, Reference data: Spanish population	Direct correlation particularly in the age groups of 5 to 10, and 10 to 15 years (r = 0.251, r = 0.228; p-value< 0.001 respectively).
[Porter and Barrett, 2004] [10]	South Asian children living in UK	24 South Asian and 71 white children, <16 years, 1990 to 2002, Reference data: United Kingdom growth standards	No correlation. r = 0.067; p-value>0.5.
[Dayal et al., 2016] [21]	North Indians living in India	467, <16 years, 2005 to 2014, Reference data: WHO reference data	No Correlation. r = 0.010; p-value = 0.82.
[Derraik et al., 2012] [19]	New Zealanders living in Auckland region	884, <15 years, 1990 to 2009, Reference data: British 1990 Growth Reference Data	No correlation. Value for r is not reported.
[O’Connell et al., 2007] [15]	Children from Australia.	1112, <20 years, 1992 to 2003, Reference data: UK 1990 growth charts as no similar data was available for Australia.	No correlation. r = 0.01, p-value = 0.7.
[Dabelea et al., 2006] [13]	Largely non-Hispanic white children from U.S.A.	449, <20 years, 2001 to 2004, Reference data: National Center for Health Statistics.	Inverse correlation seen only in those youth with reduced beta-cell function. (Regression coefficient: −7.9, p-value = 0.003).
[Evertsen et al, 2009] [17]	Largely Caucasians children from Southeastern Wisconsis, USA	1618, <19 years, 1995 to 2004, Reference data: 2000 Centers for Disease Control and Prevention (CDC) growth charts.	Inverse correlation. Value for r is not reported. Statistics is based on F-statistic one-way ANOVA.
[Knerr et al. 2005] [12]	European children from Germany and Austria- Central Europe.	9248, <20 years, 1990 to 2003, Reference: Multicenter surveys carried out over a comparable time-span throughout Germany.	Inverse correlation. Value for r is not reported.
[Kibirige et al. 2003] [9]	White (Anglo-Saxon) children from United Kingdom.	94, <16 years, 1980 to 2000, Reference data: 1990 U.K. growth standards	Inverse correlation. r = -0.39; p-value < 0.001.
[Betts et al., 2005] [11]	Caucasian (Anglo-Saxon) children from United Kingdom.	168, <16 years, 1980 to 2002, Reference data: 1990 UK reference standards	Inverse correlation. (r = -0.30, p-value < 0.001).
This study.	Kuwaiti native children of Arab ethnicity.	602, 2 to 18 years, 2011 to 2013, Reference data: Kuwaiti pediatric population recorded during comparable time-period	Inverse correlation in children and adolescents of age 6 to 18 years. (r = -0.28, p<0.001); (r, in case of children with BMI > national average = -0.38, p-value<0.001).

Results from our study indicate significant inverse correlation between BMI z-scores and age at onset of T1D in native Kuwaiti children diagnosed at 6 to 18 years of age. Our observation of inverse correlation is in line with those on populations from USA, UK, and Central Europe [9,11–13,17], but differ from those populations in which direct (Spain) [14] or no (India, Australia, and New Zealand) [10,15,19,21] relationships were observed.

The overall correlation of r = -0.28 found in our study was comparable with the study of Betts et al. [11] that reported inverse correlation between BMI z-score and age at onset. In order to further review the consistencies between the estimates on Pearson correlation coefficient, we evaluated the significance of differences between the derived coefficient from our study (r = -0.28) and the Pearson coefficients reported in the other studies–note: not all the listed studies reported Pearson correlation coefficients. Comparison with the study of Gimenez et al. [14], which reported direct correlation, revealed significant difference in the estimate for relationship (p-value = 0.0). Comparison with the studies of Porter and Barrett [10], Dayal et al. [21], and O’Connell et al. [15], all three of which reported absence of correlation, revealed significant differences in the estimate for relationship (p-value = 0.0019, 0.00, and 0.00, respectively). Comparison with the studies of Kibirige et al. [9], and Betts et al. [11], both of which reported inverse correlation, revealed non-significant differences in the estimate for relationship (p-value = 0.2784, and 0.8098, respectively). Thus we find that the estimate for the relationship in our study was significantly different from that listed in studies reporting direct or no correlation but was not significantly different from that listed in studies reporting inverse correlation.

The studies listed in Table 1 have examined the generalization of the accelerator hypothesis (on the role of weight gain in childhood diabetes) in various populations–the presented results point to heterogeneity in the direction of correlation between BMI z-score and onset age. In order to provide scientific evidence that the observed variation in the association between populations is not an artifact of possible variations in statistical power and study designs of the studies, a systematic overview of the studies is as presented below:

1. Except the study of Porter and Barret [10], that reports absence of correlation in South Asian children living in UK, all other studies are sufficiently powered–they have a power of 80% to detect small effect size of r = 0.20–0.30 at the conventional alpha level of 0.05. It is worth pointing out the sample sizes of exemplary studies: (a) Direct correlation: sample size of 3203 in the study of Gimenez et al. [14]; (b) No correlation: sample size of 1112 in the study of O’Connell et al. [15]; and (c) Inverse correlation: sample sizes of 1618, and 9248 in the studies of Evertsen et al. [17] and Knerr et al. [12], respectively.
2. All the listed studies utilize retrospectively reviewed data and hence are uniformly of cross-sectional study design. These studies, (except our study and that of Dabelea et al. [13]) in general have long recruitment period. These studies are uniformly restricted to considering obesity-related anthropometric traits except the study of Dabelea et al. [13] which includes data on loss in beta cell functionality. Pearson correlation tests are used more often than linear regression or ANOVA tests. Almost all the studies consider national growth reference data (or growth charts) to calculate BMI z-scores; the only two exceptions are the studies on New Zealanders and Australians (O’Connell et al. [15] and Derraik et al. [19]) that used British 1990 Growth Reference Data; these two studies reported absence of correlation; however, Table 1 lists other studies that reported absence of correlation.
3. The cohorts considered in all the studies are uniformly comprised of children and young adolescents. Mean age at onset in studies utilizing cohorts of similar age range is similar irrespective of the type of observed correlation: (a) mean onset age in cohorts comprising children with onset age <16 years is uniformly around 7.5 years in studies that report either absence of correlation (Porter and Barrett [10]: 7.8 years; Dayal et al. [21]: 7.27 years; and Derraik et al. [19]: 7.6 years) or inverse correlation (Kibirige et al. [9]: 7.5 years; and Betts et al. [11]: 8.1 years). (b) Mean onset age in cohorts comprising children with onset age <20 years is uniformly around 9.5 years in studies that report either absence of correlation (O’Connell et al. [15]: 9.47 years) or inverse correlation (Dabelea et al. [13]: 9.6 years; our study: 9.1 years upon considering the complete cohort of 2–18 years).
4. Correlations are observed in general in the entire cohort though the extent of correlations can differ in certain subgroups such as in (a) Gimenez et al. [14]: the observed direct correlations are particularly strong in the age groups of 5–10 and 10–15 years; and (b) the observed inverse correlation in our study is seen only in children and adolescents of age 6 to 18 years but not in <6 years age group.
5. The studies where no correlation was observed mention obesity as not necessarily the only factor associated with early onset of T1D. In this context, it is interesting to note from the study of Dabelea et al. [13] that inverse correlation is seen only in those youth with reduced beta-cell function; however, BMI has been shown as an important driver of beta-cell loss in T1D [41].

The overview as presented above does not lead to a conclusion that the observed heterogeneity in the direction of relationship among the different populations is a result of heterogeneity in study power and design. Ethnicity dependence in risk factors and associations among them is well-documented in literature—for example, we demonstrated earlier that T2D risk assessments developed using regional data outperform generalized global assessment tools (such as that of American Diabetes Association) [7].

Growth charts based on national statistics are often used by health professionals to see whether a child’s weight falls into a healthy range for the child’s ethnicity, height, age, and sex. It is clinically interesting to examine how children of BMI greater than the national average (for similar ethnicity, age and sex) perform in terms of T1D onset. For this purpose, we chose BMI z-score = 0 as threshold to partition the cohort and examine the change in the extent of correlations between BMI z-score and age at onset; the z-score reflects the relative deviation from the average value (*i*.*e*. positive or a negative sign shows whether the child's actual BMI is more than the national average BMI or less than the average BMI). Upon examining the two subgroups, it was seen that the mean onset age in the group of children with positive BMI z-score is lower (p-value = 0.0001) than the group having negative BMI z-score (Fig 2); the values for mean onset age for the two groups are 9.6±2.4 and 10.6±2.7; p-value = 0.0001, respectively. The rate of decline in onset age is more steep (p-value = 0.04) in children having positive BMI z-score. Upon further examining the cohort using BMI z-score quartiles (S1 Table), it was seen that the mean onset age in the fourth quartile is significantly lower (p-value = 2.316e-05) than that seen in the first quartile–the mean onset age in the first and fourth quartiles were 10.9±2.7 (p-value = 1.567e-05), and 9.4±2.4 (p-value = 1.469e-07), respectively. The correlation coefficient for the fourth quartile was higher than that for the first quartile (-0.46 versus -0.39) though the difference was not statistically significant.

The above discussed results that the inverse association got further augmented when children of BMI higher than the national average were considered (r = -0.38) but diminished (still maintaining significant associations) when children of BMI lower than the national average were considered (r = -0.19); thus the study illustrated that age at diagnosis of T1D in children is indeed a function of body mass (Fig 2). We have earlier shown that in the case of T2D as well, BMI is inversely associated with age at diagnosis [8]. Thus, it appears that the accelerator hypothesis is valid in both T1D and T2D in the Kuwaiti population.

The data showed girls were diagnosed with T1D at a significantly younger age than boys (9.9 and 10.6 years, respectively; p<0.01). The difference in age at diagnosis between sexes remained statistically significant (p-value = 0.014) when corrected for BMI z-scores in the regression model. This indicates that obesity affects the tempo of T1D presentation differently in girls compared with boys. A similar observation has been reported in other populations as well; early occurrence in girls has been attributed to earlier puberty in females, with associated endocrine and metabolic changes affecting insulin resistance [40].

A potential limitation of the presented study is the relatively small sample size compared with some other studies in the literature; however, the study had sufficient power to show a correlation of r = -0.28 with 80% power at 95% certainty. Further, we did not find any significant correlation between BMI and age at onset in the age group of 2 to 6 years (r = -0.029, p-value = 0.74). However, it should be mentioned that we lack national reference data for this age group and this limitation might affect the association analysis. Nevertheless, Gimenez et al. [14] did not find significant correlation in the age group of 2 to 5 years either, while they found significant associations in age groups of 5 to10 and 10 to 15 years.

## Conclusion

A literature survey on the association between BMI and age at onset of T1D in children and adolescents conclude that populations vary in terms of support for the ‘accelerator hypothesis’. The native Arab population from Kuwait falls in the category of supporting the hypothesis–onset age of T1D is accelerated in heavier children. It is required to test the hypothesis in further ethnic minorities and in different age groups to bridge the two ends–childhood and adulthood diabetes.

## Supporting information

S1 Dataset Minimal Dataset listing for each participant of the cohort the sex, age at onset of T1D, and BMI z-score at onset of T1D. (CSV)

S1 Table Mean onset age of Type 1 diabetes by BMI z-score quartiles. (DOCX)
