# Supplementary material for: Ethnic differences in association of high body mass index with early onset of Type 1 diabetes – Arab ethnicity as case study
Source: PLoS One. 2017 Apr 13;12(4):e0175728. doi: 10.1371/journal.pone.0175728 (PMC5391107; doi:10.1371/journal.pone.0175728)
Supplement: S1 Table — (DOCX) [file pone.0175728.s002.docx]

**S1 Table. Mean onset age of Type 1 diabetes by BMI z-score quartiles.**

| BMI Z score quartiles (and end point in terms of BMI z-score), and sample size. | Correlation coefficient, r (BMI z score * onset age) | p-value | Onset age Mean±SD**^&^** |
| --- | --- | --- | --- |
| 1^st^ Quartile (-3.06 to -1.25)  N=119 | -0.39 | 1.567e-05 | 10.9±2.7 |
| 2^nd^ Quartile (-1.24 to -0.44)  N=118 | 0.03 | 0.79 | 10.6±2.7 |
| 3^rd^ Quartile (0.39)  (-0.43 to 0.39)  N=118 | 0.06 | 0.51 | 10.0±2.5 |
| 4^th^ Quartile (3.52)  (0.40 to 3.52)  N=119 | -0.46 | 1.469e-07 | 9.4±2.4 |

**^&^**p-value for difference in mean onset age between first and second quartiles = 0.4604; between first and third quartiles = 0.01315; and between first and fourth quarties – 2.316e-05.
